# Cutting Costs and Carbon: Re-evaluating Routine Group and Save Testing for Appendicectomies

**DOI:** 10.7759/cureus.94781

**Published:** 2025-10-17

**Authors:** Obed Amoako-Adjei, Alexia Defer, Bishow Karki, Ananya Nayak

**Affiliations:** 1 General Surgery/Trauma and Orthopaedics, Mid Yorkshire NHS Teaching Trust, Wakefield, GBR; 2 General Surgery, Mid Yorkshire NHS Teaching Trust, Wakefield, GBR; 3 General Surgery, Suture Centre, Hull University Teaching Hospital, Leeds, GBR

**Keywords:** appendicectomy, carbon footprint, efficiency, group and save, laparoscopic, preoperative testing, sustainability, transfusion

## Abstract

Background: Routine preoperative Group and Save (G&S) testing is traditionally performed before appendicectomy to ensure blood availability; however, transfusion is exceedingly rare in this context. In an era of value-based and sustainable healthcare, this study re-evaluates the necessity of routine G&S testing by analyzing its clinical utility, cost, and environmental impact in a district hospital in the United Kingdom (UK).

Methods: A retrospective review of 265 appendicectomies performed between December 1, 2023, and June 30, 2024, at Pinderfields Hospital was conducted. Demographic, biochemical, and intraoperative data were collected, including ASA (American Society of Anesthesiologists) grade, white cell count (WCC), and C-reactive protein (CRP). Cases were categorized as uncomplicated or complicated based on operative findings. Independent-samples t-tests were used to compare continuous variables (age, WCC, CRP), and chi-square tests assessed associations between ASA grade and case complexity. The 95% confidence interval (CI) for the zero-transfusion rate was calculated using the Clopper-Pearson exact method. Financial and carbon estimates were derived from published UK data.

Results: Of the 265 cases, 261 (98.5%) were laparoscopic, with four conversions to open surgery. No intraoperative or immediate postoperative transfusions occurred (transfusion rate 0%; 95% CI 0-1.4%). Patients with complicated appendicitis had significantly higher mean WCC (15.2 vs 11.9 ×10⁹/L; p < 0.001), CRP (115.8 vs 57.3 mg/L; p < 0.001), and age (41.7 vs 32.6 years; p = 0.001). Higher ASA grades were also associated with increased case complexity (χ²(3) = 13.07, p = 0.004). A total of 520 G&S samples were submitted, of which 78 (15%) were rejected. The estimated testing cost was £7,735 for the study period (£14,116 annually), and the carbon footprint was 190.06 kg CO₂e.

Conclusions: Routine preoperative G&S testing for appendicectomy provides negligible clinical benefit while incurring measurable financial and environmental costs. Elevated inflammatory markers, higher ASA grades, and older age were predictive of complicated disease, supporting a selective, risk-based testing strategy. Restricting G&S to higher-risk patients could maintain safety, enhance efficiency, and contribute to National Health Service (NHS) sustainability goals.

## Introduction

Acute appendicitis is among the most common surgical emergencies worldwide, with laparoscopic appendectomy being one of the most frequently performed emergency general surgical procedures [1]. Preoperative Group and Save (G&S) testing is routinely performed in many surgical procedures to ensure blood availability in case of haemorrhage. However, intraoperative blood loss during appendicectomy requiring blood transfusion is uncommon, which questions the necessity of routine G&S testing in this context [2].

The healthcare sector is increasingly recognizing its responsibility in mitigating climate change, given its significant carbon footprint and vulnerability to climate-driven health impacts. In the United Kingdom, the National Health Service (NHS) has committed to achieving net-zero emissions, with pathology and laboratory services identified as key contributors. Even small reductions in unnecessary testing can lead to meaningful environmental and economic gains [3-5].

This audit evaluates the clinical utility, financial implications, and environmental impact of routine G&S testing prior to laparoscopic appendectomy, supporting a change in practice at Mid Yorkshire NHS Trust.

This article was presented as an abstract poster at the 2025 Association of Surgeons in Training (ASiT) Conference on March 8, 2025, in Belfast, United Kingdom.

## Materials and methods

A retrospective review of 265 appendectomies performed between December 1, 2023, and June 30, 2024, at Pinderfields Hospital was undertaken. Details of all appendicectomies done within the study period were extracted from Theatreman Aqua, which is the software used in the operating theatres of Pinderfields Hospital. All emergency and interval appendicectomies performed from December 2023 to June 2024 in all age groups were included. Appendicectomies performed at the time of other surgeries, such as hysterectomies and colectomies, were excluded before data collection, as such patients have a different surgical risk profile, undergo higher-risk surgeries for different morbidities such as cancer, and have greater potential for blood loss.

Specific data for individual cases were then extracted from operative notes and perioperative records on CITO and PPM Plus patient electronic record systems. All patient records used were anonymized before data analysis.

Demographic data collected included patient age, gender, and American Society of Anesthesiologists (ASA) grade. The surgical approach (open or laparoscopic) and, for open procedures, the type of incision used were recorded. Biochemical parameters, including preoperative haemoglobin, white cell count (WCC), and C-reactive protein (CRP) levels, were documented for each patient. Data regarding case complexity, based on intraoperative findings, and any additional procedures performed concurrently were also noted. In addition, the number of Group and Save (G&S) samples submitted, sample rejections (including reasons for rejection), resubmitted samples, and any intraoperative or postoperative blood transfusion events were recorded.

The average cost of raw materials used for G&S sample testing was provided by the transfusion laboratory lead. Specific kit or manufacturer details were not available, as the test is performed using standard in-house laboratory procedures. Additional cost estimates were obtained from published UK literature [6]. Carbon emissions per sample were calculated using literature estimates, including the footprint of phlebotomy, sample transport, and laboratory processing [7].

Data analysis was performed using Microsoft Excel (Microsoft Corporation, Redmond, Washington) and IBM SPSS Statistics for Windows, Version 27 (Released 2020; IBM Corp., Armonk, New York). Descriptive statistics were used to summarize demographic and biochemical data. Continuous variables (age, WCC, CRP) were compared between uncomplicated and complicated appendicitis groups using independent-samples t-tests assuming unequal variances. Associations between categorical variables (ASA grade and case complexity) were assessed using Chi-square tests. Statistical significance was defined as p < 0.05. The 95% confidence interval for the zero-transfusion rate was calculated using the Clopper-Pearson exact binomial method.

This study involved a retrospective analysis of anonymized patient records. In accordance with UK Health Research Authority (HRA) guidelines, formal ethical approval and individual patient consent were waived, as this was a service audit. Institutional approval was obtained from Mid Yorkshire NHS Trust.

## Results

Out of 265 appendectomies, 261 (98.5%) were laparoscopic and four (1.5%) were converted to open procedures. Of the four laparoscopic appendicectomies that required conversion to open surgery, two were completed via a standard midline laparotomy and two via a lower midline incision. Among these cases, one involved a right hemicolectomy with ileocolic anastomosis, one required an ileocolic resection, and the remaining two were completed as standard appendicectomies. These conversions reflect the technical complexity in selected cases and support the consideration of a selective G&S approach.

There were 135 (51%) females and 130 (49%) males. The median age of patients included in this study was 32 years (range, 6-86 years). Table 1 demonstrates the age distribution of cases included in this study.

**Table 1:** Age Distribution

Age Group	Number of Patients (%)
Less than 16 years	49(18.5)
16-60	183 (69)
Above 60	33(12.5)

Most patients were ASA grades I and II (94%), reflecting a generally low-risk surgical population. Table 2 elaborates the ASA grade distribution.

**Table 2:** ASA Grade ASA: American Society of Anesthesiologists physical status classification. I: Healthy patient. II: Mild systemic disease. III: Severe systemic disease. IV: Severe systemic disease that is life-threatening.

ASA Grade	Number of Patients (%)
I	92 (35)
II	157 (59)
III	15 (5.6)
IV	1 (0.4)

Preoperative inflammatory markers showed variable responses across the cohort (Table 3). The mean white cell count (WCC) was 12.9 ± 5.1 ×10⁹/L. Categorically, 46.4% of patients had a normal WCC (4-12 ×10⁹/L), while 53.6% had an abnormal WCC (<4 or >12 ×10⁹/L). The mean C-reactive protein (CRP) was 90.2 ± 95.1 mg/L. CRP was normal (≤10 mg/L) in 31.7% of patients, moderately elevated (11-100 mg/L) in 42.3%, and markedly elevated (>100 mg/L) in 26.0%, indicating that a substantial proportion of patients presented with significant systemic inflammation.

**Table 3:** Pre-operative WCC and CRP WCC: white cell count, CRP: C-reactive protein.

Parameter	Category	Number of Patients (%)
WCC (×10⁹/L)	4-12 (normal)	123 (46.4)
	<4 OR >12 (abnormal)	142 (53.6)
CRP (mg/L)	≤10 (normal)	84 (31.7)
	11-100	112 (42.3)
	>100	69 (26)

Preoperative haemoglobin was lower than normal in 32 cases (10.2%), using reference ranges of 130-180 g/L for male and 115-165 g/L for female. Table 4 shows the gender distribution of low haemoglobin.

**Table 4:** Pre-operative Haemoglobin Range of normal haemoglobin used: male, 130–180 g/L; female, 115–165 g/L). hb: haemoglobin.

Pre-operative Haemoglobin	Frequency (%)
Males with hb <130	21 (7.9)
Females with hb < 115	11 (4.1)

The spectrum of intraoperative findings, including appendiceal masses, perforation, and severe inflammation, as shown in Table 5, underscores the value of preoperative assessment through examination and imaging to identify cases likely to be technically complex. Targeted G&S testing in such patients can optimize preparedness for procedures that may involve significant blood loss, such as hemicolectomies.

**Table 5:** Case Complexity Based on Intra-operative Gross Pathology

Gross Appearance of the Appendix	Frequency (%)
Inflamed	163 (61.5)
Perforated/gangrenous	68 (25.6)
Normal	24 (9.2)
Appendix mass	10 (3.7)

For the purpose of analysis, normal and inflamed appendices were classified as uncomplicated, while perforated, gangrenous, and appendiceal mass cases were classified as complicated. Independent-samples t-tests were performed to compare age, CRP, and WCC levels between the uncomplicated and complicated groups. A chi-square test was used to assess the association between ASA grade and case complexity.

The mean preoperative WCC was significantly higher in patients with complicated appendicitis (15.2 ×10⁹/L, SD 5.4) compared with uncomplicated cases (11.9 ×10⁹/L, SD 4.7; t(108) = −4.44, p < 0.001). Similarly, the mean CRP level was significantly higher in the complicated group (115.8 mg/L, SD 106.9) than in uncomplicated cases (57.3 mg/L, SD 66.9; t(101) = −4.23, p < 0.001). Patients with complicated appendicitis were also significantly older (mean 41.7 years, SD 20.0) than those with uncomplicated disease (mean 32.6 years, SD 17.2; t(107) = −3.34, p = 0.001).

Analysis of ASA grades demonstrated a significant association between higher ASA class and case complexity (χ²(3, N = 256) = 13.07, p = 0.004), indicating that patients with greater systemic comorbidity were more likely to have complicated intraoperative findings. No patients required intraoperative or immediate postoperative blood transfusion. The observed transfusion rate was therefore 0% (95% CI 0-1.4%, Clopper-Pearson method), confirming the very low risk of haemorrhage in both uncomplicated and complicated appendicitis.

Collectively, these results suggest that elevated inflammatory markers (WCC, CRP), higher ASA grades, and older age are predictive of technically challenging appendicectomies. Incorporating these factors into preoperative assessment may support a selective, risk-based approach to G&S testing, ensuring laboratory resources are reserved for patients with higher procedural risk.

The Trust’s protocol required two G&S samples per patient without prior results and one sample for patients with pre-existing results. A total of 520 G&S samples were submitted during the study period. Of these, 78 samples (15%) were rejected. The majority of rejections were due to labelling errors, while a proportion occurred because a valid existing G&S result was already present. It is important to note that the hospital’s electronic system only flags existing results once they are uploaded; therefore, multiple samples may occasionally be sent by different staff members without realizing that a sample for the same patient is already being processed. Table 6 summarizes the reasons for sample rejections and the number of samples attributed to each cause.

**Table 6:** Reasons for Sample Rejection DOB: date of birth; NHS: National Health Service; G&S: Group and Save.

Reason	n
Wrong/expired bottle	4
Clerical errors – incorrect name/DOB/NHS number/no labelling/request not signed	37
Previous sample present	32
Insufficient sample	3
Sample taken less than 15 minutes apart from the initial sample	2

A total of 442 (85%) samples sent were processed by the laboratory. No intraoperative or immediate postoperative transfusions occurred. One patient received two units of blood one month postoperatively due to unrelated complications.

The cost of G&S testing is estimated to be £17.5 per test based on published literature [6]. Table 7 outlines further costing details and the total cost incurred for processing the number of tests in this study. The estimated carbon emission per test is 0.43 kgCO₂e [7], resulting in a total emission of 190.06 kgCO₂e for 442 samples.

**Table 7:** Cost of G&S Testing G&S: Group and Save.

Item	Unit Cost	Total Cost for 442 Samples
Local G&S Testing (excluding personnel and utilities)	£1.91	£844.22
Estimated cost of G&S Testing	£17.50	£7,735
Standard red cell unit	£183.78	N/A

## Discussion

Necessity of G&S testing

The absence of perioperative transfusion, with a calculated 95% CI of 0-1.4%, highlights the minimal bleeding risk associated with appendicectomy, even among patients with complicated disease. When interpreted alongside the significant associations between elevated inflammatory markers, higher ASA grades, and increased case complexity, these findings support a selective preoperative Group and Save policy. Patients with high WCC or CRP, ASA ≥ III, or imaging evidence of appendiceal mass may warrant testing, whereas those with low-risk profiles could safely forgo it, thereby optimizing both patient safety and resource utilization. These findings align with previous studies showing that laparoscopic appendicectomy is a low-bleeding-risk procedure, with transfusion rates approaching zero [2,8,9]. This reinforces the argument that routine preoperative testing may be unnecessary in this context.

A more selective approach, reserving G&S testing for patients with anaemia, coagulopathy, or other comorbidities, could maintain patient safety while avoiding unnecessary interventions. Tailoring testing to individual risk factors aligns with the principles of precision medicine and evidence-based care, ensuring that resources are allocated where clinically justified. This approach has been recommended by other appendicectomy studies [2,10].

Case complexity can often be anticipated preoperatively through assessment of laboratory markers (WCC, CRP), ASA grade, and imaging or clinical findings suggestive of severe inflammation or appendiceal mass. Additional factors such as previous abdominal surgery and elevated BMI may also influence operative difficulty; however, data on these variables were not available in this study. Patients with markedly elevated inflammatory markers, higher ASA grades, or radiological evidence of complicated appendicitis are more likely to require technically demanding procedures such as ileocolic resection or right hemicolectomy, which carry an inherently higher risk of blood loss. Implementing selective G&S testing in such cases provides a safety net for potential transfusion needs, while avoiding unnecessary testing in low-risk patients. This approach ensures a balance between clinical safety, cost-effectiveness, and resource stewardship.

Financial burden

While the per-sample cost of G&S testing may appear modest (£17.50 per sample based on literature estimates), the cumulative financial burden is substantial when scaled across hundreds of procedures. In this study, 442 samples were processed, resulting in an estimated total cost of £7,735 for the study period and an estimated £14,116 annually. This figure excludes additional indirect costs such as staff time and the cost of consumables for phlebotomy. The large number of rejected samples represents additional workload and further indirect costs. These inefficiencies not only increase expenditure but also divert resources from higher-value clinical activities.

Other studies have similarly highlighted the financial impact of low-value preoperative testing [6,8,10]. By reducing unnecessary G&S testing, hospitals can achieve meaningful cost savings that could be redirected to other areas of patient care, improving overall efficiency and resource utilization.

Environmental impact

The carbon footprint of healthcare services is increasingly recognized as a critical concern. Laboratory testing, in particular, contributes disproportionately to hospital-based carbon emissions due to energy-intensive processes, transport, and consumables [11-13]. In this study, G&S testing generated 190.06 kgCO₂e over the study period and is estimated to generate 325.8 kgCO₂e annually.

The cumulative environmental impact across multiple hospitals and years is substantial. Reducing unnecessary testing is therefore an important strategy for hospitals seeking to align clinical practice with the NHS Net Zero initiative. Minimizing low-value laboratory testing contributes not only to environmental sustainability but also supports broader public health objectives by mitigating the health effects of climate change.

Operational and system-wide implications

Beyond direct financial and environmental considerations, routine G&S testing imposes operational burdens. A total of 78 (15%) samples were rejected, primarily due to clerical errors and duplicate testing, highlighting inefficiencies that affect laboratory workflow and staff workload. Optimizing preoperative testing protocols can reduce avoidable errors, enhance workflow efficiency, and improve laboratory turnaround times.

This study demonstrates that clinical practice can be adapted based on local data, supporting evidence-driven policy change. Following this study, Mid Yorkshire NHS Trust revised its protocol to limit G&S testing to selected patients. No adverse events have been reported, confirming the safety and feasibility of this approach. Such locally driven audits provide a model for other hospitals aiming to balance patient safety with cost-effectiveness and sustainability goals.

Broader implications for low-risk surgery and value-based care

The principles demonstrated here can be applied to other low-risk procedures, including elective laparoscopic cholecystectomy [14,15]. Rationalizing preoperative testing represents an important component of value-based care, emphasizing interventions that provide tangible clinical benefit while minimizing waste and environmental impact.

Encouraging healthcare professionals to question routine practices and adopt sustainability-minded approaches can have broad implications for NHS policy. National guidance should reflect evolving evidence, promoting protocols that are both clinically sound and environmentally responsible. By embedding sustainability into standard care pathways, healthcare systems can make meaningful progress towards reducing unnecessary testing, lowering costs, and mitigating the sector’s carbon footprint.

This study highlights the interconnected nature of clinical decision-making, financial stewardship, and environmental responsibility. Small-scale decisions, such as whether to perform a routine G&S test, can accumulate into significant impacts across healthcare systems. By adopting selective testing protocols, hospitals can simultaneously enhance patient care, reduce avoidable expenditure, and contribute to climate goals.

These findings reinforce the concept of the “triple bottom line” in healthcare, where decisions are evaluated not only for clinical effectiveness but also for economic efficiency and environmental sustainability [11]. Integrating these considerations into routine surgical practice encourages a holistic approach to patient care that benefits patients, staff, and society at large.

## Conclusions

Routine G&S testing prior to laparoscopic appendectomy provides negligible clinical benefit while generating substantial financial and environmental costs. A selective, risk-based approach improves operational efficiency, reduces waste, and supports NHS sustainability goals without compromising patient safety.

Case complexity can often be anticipated using preoperative markers such as WCC, CRP, ASA grade, and imaging or clinical findings suggestive of severe inflammation or appendiceal mass. Selectively performing G&S testing for patients identified as higher risk ensures preparedness for technically challenging procedures such as ileocolic resection or right hemicolectomy, while avoiding unnecessary testing in low-risk cases.

By implementing targeted testing, hospitals can reallocate resources, including staff time, laboratory capacity, and consumables, to higher-priority clinical services. Over time, this contributes not only to cost savings but also to meaningful reductions in carbon emissions and environmental impact, aligning surgical practice with national net-zero objectives.

The findings underscore the importance of continually re-evaluating long-standing medical protocols in light of current evidence. Institutions should adopt policies that balance patient safety, cost-effectiveness, and environmental stewardship. Expanding this approach to other low-risk procedures offers an opportunity to advance value-based healthcare, promote sustainability, and serve as a model for evidence-driven practice change across the NHS.
